# Efficacy of Dysesthesia-Matched Transcutaneous Electrical Nerve Stimulation on Chronic Lower Limb Numbness in Patients With Degenerative Lumbar Spondylolisthesis: A Case Report

**DOI:** 10.7759/cureus.70209

**Published:** 2024-09-25

**Authors:** Kotaro Homma, Yuki Osaka, Himito Okazaki, Hidetaka Furuya, Masahiro Hoshino

**Affiliations:** 1 Department of Rehabilitation, Sonoda Third Hospital/Tokyo Medical Institute Tokyo Spine Center, Tokyo, JPN; 2 Department of Orthopaedics, Sonoda Third Hospital/Tokyo Medical Institute Tokyo Spine Center, Tokyo, JPN

**Keywords:** dysesthesia, lumbar degenerative spondylolisthesis, numbness, spine, transcutaneous electrical nerve stimulation

## Abstract

Degenerative lumbar spondylolisthesis (DLS) is a degenerative condition that causes leg pain, numbness, and gait disturbances. Despite surgical treatment, reducing numbness is more challenging than reducing pain, and there is currently no established effective intervention method. Dysesthesia-matched transcutaneous electrical nerve stimulation (DM-TENS) is a novel technique that adjusts TENS settings to match the sensation of numbness, potentially improving it. We applied DM-TENS to a post-operative DLS patient with chronic lower limb numbness. As a result, the NRS for numbness was 8.00 before the intervention but improved to 6.67 ± 0.47 after HF-TENS and to 1.67 ± 0.47 after DM-TENS (mean ± SD), indicating that DM-TENS was more effective in reducing chronic lower limb numbness. This report highlights the promising potential of DM-TENS as an effective intervention for reducing numbness in DLS patients, who often experience lower limb numbness.

## Introduction

Degenerative lumbar spondylolisthesis (DLS) is one of the most frequently treated degenerative conditions in orthopedics [1], characterized by the anterior slip of an upper vertebra over a lower one, leading to compression of the cauda equina and spinal nerve roots, and causing stenosis [2]. DLS has been reported to cause low back pain, leg pain, and gait disorders, thereby worsening the quality of life (QOL) of affected patients [3]. While surgical treatment has been shown to yield more favorable outcomes than non-surgical approaches [4], numbness often persists post-operatively compared to leg pain [5]. Moreover, numbness has been suggested to impact postoperative QOL and patient satisfaction [6,7]. Currently, pharmacological therapy is widely used for numbness, but its effectiveness is limited, and adverse events have been reported [8].

Recently, Nishi et al. [9] reported a new intervention for numbness called dysesthesia-matched transcutaneous electrical nerve stimulation (DM-TENS), which adjusts the intensity and frequency of electrical stimulation to match the subject's sensation of numbness. This method has shown efficacy in relieving upper limb numbness in patients with spinal cord injuries and strokes. In this report, we demonstrate the effectiveness of a novel electrical stimulation technique, DM-TENS, as an intervention to improve chronic lower extremity numbness in DLS subjects.

## Case presentation

Case introduction

**Figure 1:**
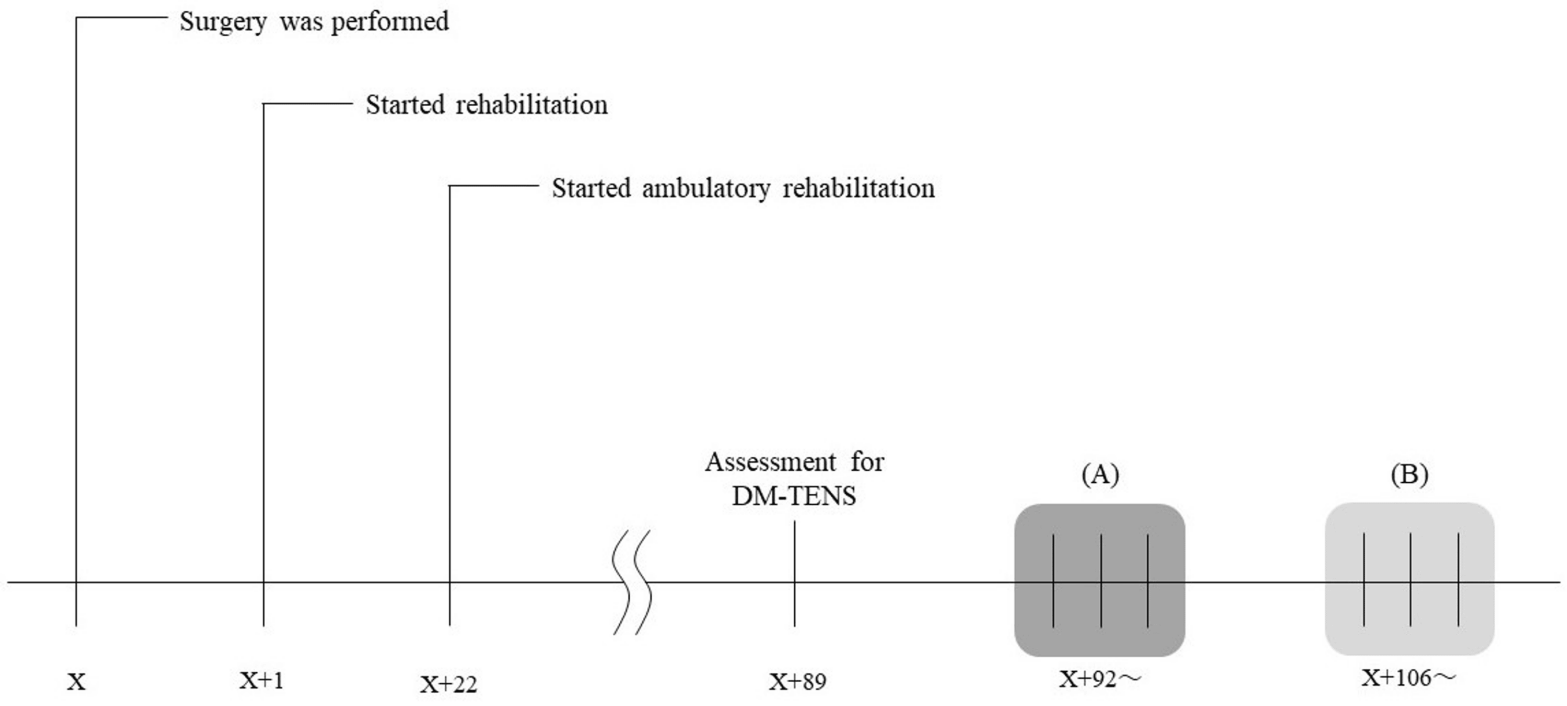
Timeline from the surgery Timeline from the surgery to the start of DM-TENS treatment. X indicates the day of surgery. (A) Involved HF-TENS combined with standard physical therapy, while (B) involved DM-TENS combined with standard physical therapy. DM-TENS: dysesthesia-matched transcutaneous electrical nerve stimulation; HF-TENS: high-frequency transcutaneous electrical nerve stimulation.

The patient was a 60-year-old female who had been experiencing low back pain and leg numbness for more than 10 years. She was diagnosed with DLS (L4) and underwent extreme lateral interbody fusion (XLIF) at L4/5 on day X. The timeline from the surgery to the start of outpatient care and the intervention is shown in Figure 1. At the start of the intervention, despite improvement in low back pain, numbness with a Numeric Rating Scale (NRS) score of 8 was observed in the plantar region. On day X+89, she was independent in activities of daily living and used a T-cane for walking, similar to her pre-operative state. She could do household tasks, but the numbness made long activities difficult, requiring frequent breaks and disrupting her daily life. Also, the patient did not exhibit significant muscle weakness, neurologic claudication, or abnormal reflexes that would interfere with daily life, with only numbness persisting. The patient was a 60-year-old female who had been experiencing low back pain and leg numbness for more than 10 years. She was diagnosed with DLS (L4) and underwent XLIF at L4/5 on day X. The timeline from the surgery to the start of outpatient care and the intervention is shown in Figure 1. At the start of the intervention, despite improvement in low back pain, numbness with a NRS score of 8 was observed in the plantar region. On day X+89, she was independent in activities of daily living and used a T-cane for walking, similar to her pre-operative state. She could do household tasks, but the numbness made long activities difficult, requiring frequent breaks and disrupting her daily life. And also, the patient did not exhibit significant muscle weakness, neurologic claudication, or abnormal reflexes that would interfere with daily life, with only numbness persisting.

The Neuropathic Pain Symptom Inventory (NPSI), which reflects neuropathic pain such as numbness, showed the following average scores: superficial spontaneous: 0.00, deep spontaneous: 7.00, paroxysmal: 4.00, evoked: 6.67, and paresthesia/dysesthesia: 10.0. The NPSI consists of 12 items and five subscales related to superficial spontaneous pain, deep spontaneous pain, paroxysmal pain, evoked pain, and paresthesia/dysesthesia [10]. The Short-Form McGill Pain Questionnaire version-2 (SF-MPQ2), which reflects pain, including neuropathic pain, showed the following average scores: continuous pain: 0.83, intermittent pain: 3.50, neuropathic pain: 2.50, and affective descriptors: 1.50. The SF-MPQ2 consists of 22 items and four subscales related to continuous pain, intermittent pain, neuropathic pain, and affective descriptors [11]. Both assessments are scored from 0 (no pain) to 10 (maximum pain level), with lower scores indicating less impairment. The disease-specific Zurich Claudication Questionnaire (ZCQ) showed the following average scores: symptoms: 4.57, function: 2.80, and satisfaction: 3.78. The ZCQ consists of 18 items and three subscales related to symptoms, function, and satisfaction, with severity scored from 1 to 5 and physical activity and satisfaction scored from 1 to 4, with lower scores indicating less impairment [12]. Cognitive function, assessed by the Mini-Mental State Examination (MMSE), was 30/30, indicating no cognitive decline.

Intervention

The patient avoided lower extremity movements and remained in a comfortable supine position. Two interventions were conducted: phase A involved high-frequency TENS (HF-TENS) combined with standard physical therapy, while phase B involved DM-TENS combined with standard physical therapy. Each intervention was performed for 30 minutes per session, once a day for three days. The standard physical therapy included a range of motion exercises and core exercises. All interventions were administered at 3:00 PM.

Dysesthesia-matched transcutaneous electrical nerve stimulation

**Figure 2:**
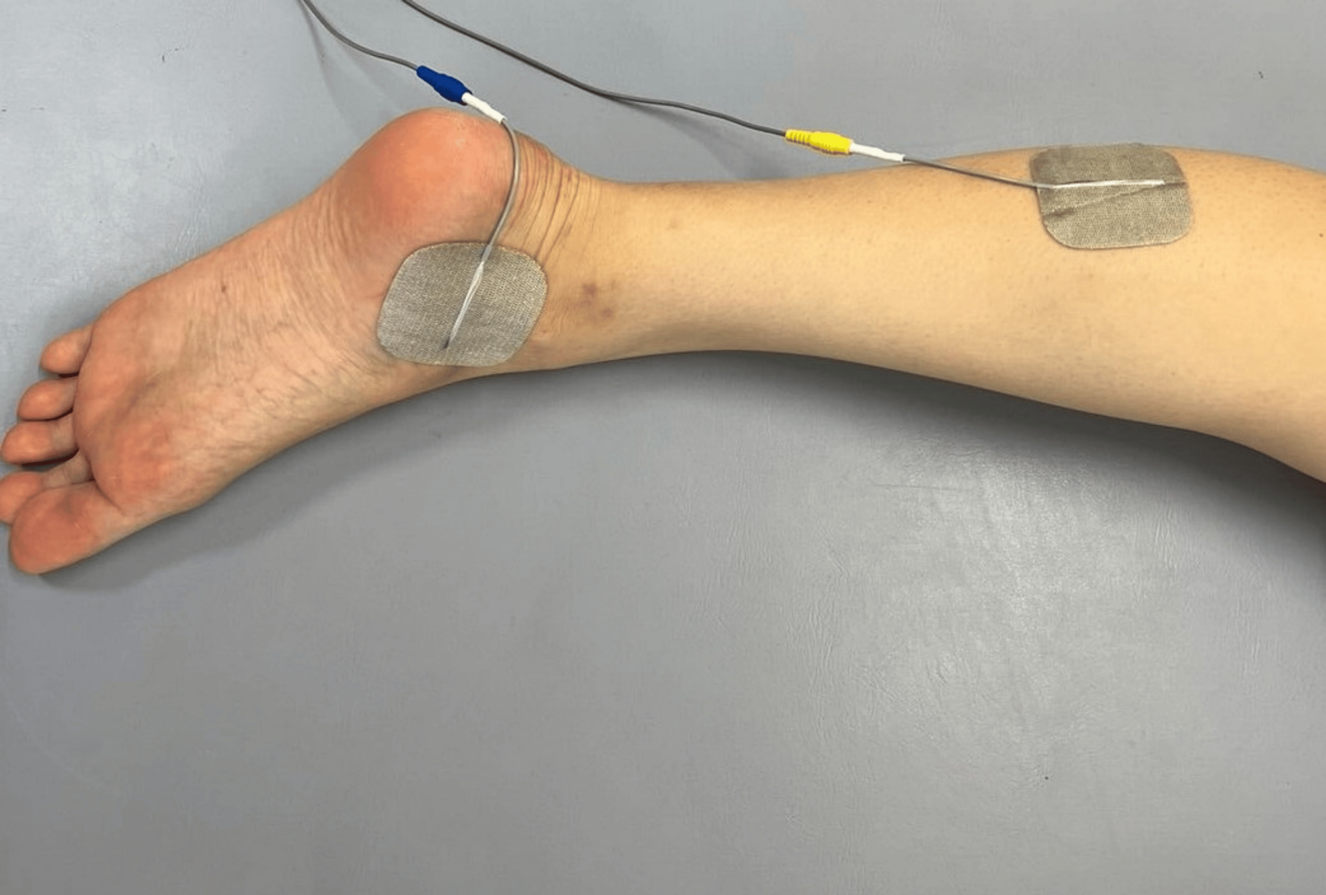
Dysesthesia-matched transcutaneous electrical nerve stimulation

DM-TENS was delivered to the plantar nerve region for 30 minutes via 25 cm² self-adhesive electrodes (Axelgaard Manufacturing, Fallbrook, CA, United States) connected to an ESTIMUS (Ito Physiotherapy and Rehabilitation Co., Tokyo, Japan) (Figure 2). DM-TENS was delivered using a continuous pulse pattern, 200 µs pulse duration, and a biphasic current with a symmetrical waveform [9].

The patient received electrical stimulation that increased in 1 mA steps at each frequency and was adjusted based on a 7-point Likert scale as follows: −3: “much stronger feeling of dysesthesia than the electrical stimulation”; −2: “feeling the dysesthesia more than the electrical stimulation”; −1: “slight feeling of dysesthesia compared to the electrical stimulation”; 0: “the same intensity of dysesthesia and the electrical stimulation but they are distinguishable”; 1: “slightly greater feeling of the electrical stimulation than the dysesthesia”; 2: “feeling the electrical stimulation more than the dysesthesia”; 3: “much stronger feeling of the electrical stimulation than the dysesthesia.” Patients were then asked to indicate whether the frequency of the electrical stimulation was lower, higher, or matched the rhythm of their spontaneous dysesthesia. To avoid bias in associating this assessment with DM-TENS, patients were not informed that this evaluation was related to DM-TENS [9]. No muscle contractions were observed during electrical stimulation.

High-frequency transcutaneous electrical nerve stimulation

HF-TENS was delivered using a continuous pulse pattern, 300 µs pulse duration, and a biphasic current with a symmetrical waveform at 100 Hz. The intensity was set at 200% of the sensory threshold and delivered for 30 minutes [9].

Evaluation and statics

The NRS was assessed before and after each intervention, and the NPSI, SF-MPQ2, and ZCQ scores were evaluated at each phase. Post-intervention NRS was visually analyzed using the Visual Aid Implying an Objective Rule (VAIOR). The VAIOR method first estimates the baseline regression line using the Theil-Sen method, then constructs a trend variability range based on the trend line and the median of absolute deviations. This variability range is then extended and projected into the intervention phase, and the proportion of data points within the intervention phase that fall below the variability range is calculated to determine the presence of an effect. When analyzing the entire dataset, the proportion of data points in the baseline phase that exceed the variability range is first calculated, and twice that value is set as the criterion. If the proportion of data points in the intervention phase that fall below the variability range exceeds this criterion, the intervention is considered effective. VAIOR is a method proposed to overcome the limitations of the commonly used visual analysis technique, the Conservative Dual Criterion (CDC) [13]. These limitations include the binary assessment of the presence or absence of immediate, progressive, delayed, or total effects and the quantification of overlap. Total scores on the NPSI, SF-MPQ2, and ZCQ were analyzed using descriptive statistics. The symptoms and function subscales of the ZCQ were analyzed using the Minimum Clinically Important Difference (MCID) [14].

Results

Table 1 shows the NRS for numbness.

**Table 1:** NRS for numbness 1st indicates first, 2nd indicates second and 3rd indicates third intervention result. Pre: pre-intervention; Post: post-intervention; HF-TENS: high-frequency transcutaneous electrical nerve stimulation; DM-TENS: dysesthesia-matched transcutaneous electrical nerve stimulation.

	1^st^		2^nd^		3^rd^	
	Pre	Post	Pre	Post	Pre	Post
HF-TENS	8	7	9	7	7	6
DM-TENS	9	2	6	1	6	2

**Figure 3:**
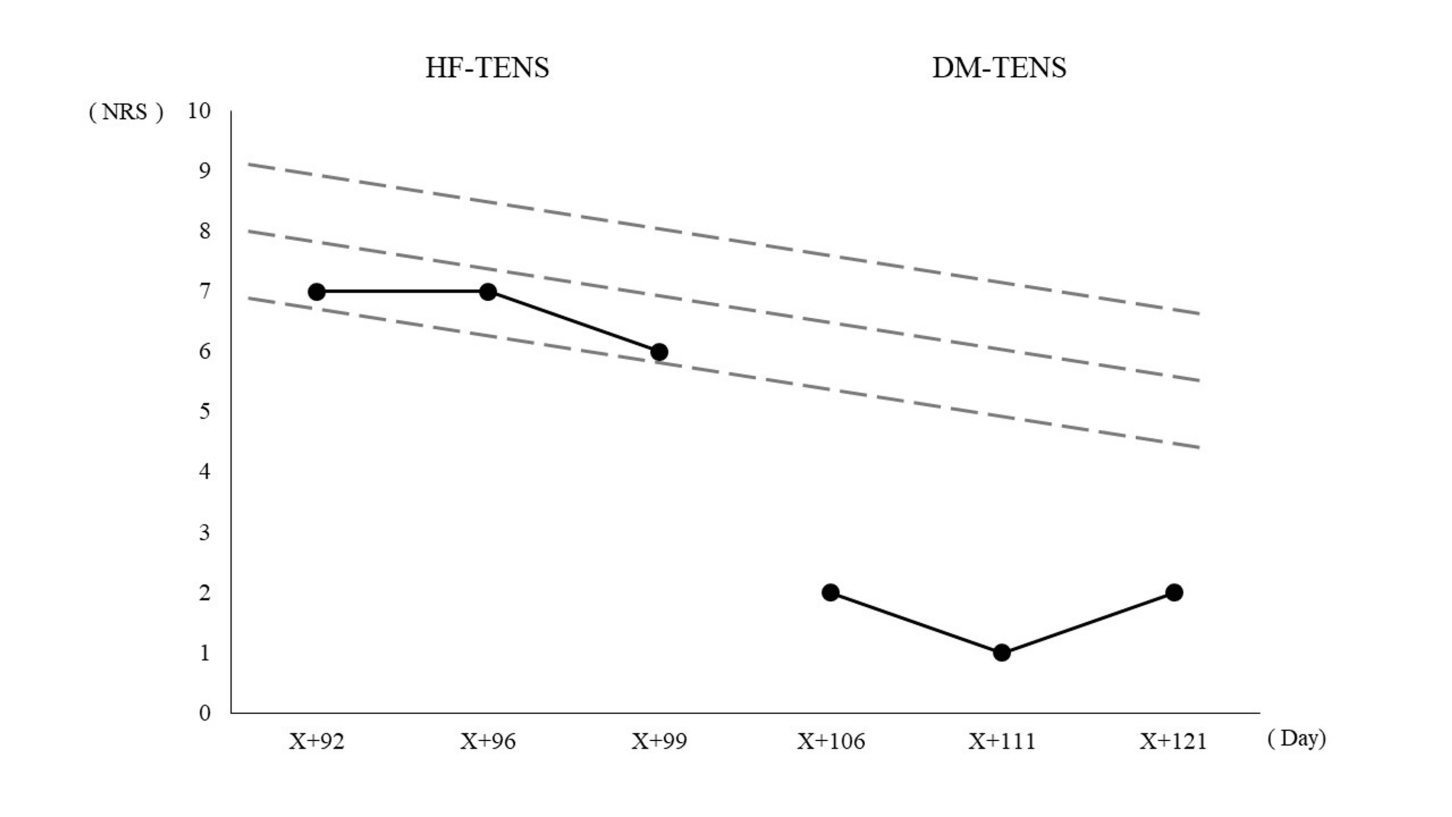
Visual analysis of NRS using a visual aid implying an objective rule The data for HF-TENS in phase A were all within the range of variation, but the data for DM-TENS in phase B exceeded the range of variation. X indicates the day of surgery. HF-TENS: High-frequency transcutaneous electrical nerve stimulation; DM-TENS: dysesthesia-matched transcutaneous electrical nerve stimulation.

The regression line was *y* = −0.5*x* + 8.50, and the trend variability range was 1. All the data in phase B were within the variability range, whereas all the data in phase A exceeded the variability range. This exceeded twice the proportion of data that exceeded the variability range in phase B, indicating that DM-TENS reduced the NRS of numbness more effectively than HF-TENS (Figure 3).

Table 2 shows the NPSI scores, Table 3 shows the SF-MPQ2 scores, and Table 4 shows the ZCQ scores.

**Table 2:** Neuropathic Pain Symptom Inventory (NPSI) results before and after each intervention The NPSI consists of 12 items and 5 subscales related to superficial spontaneous pain, deep spontaneous pain, paroxysmal pain, evoked pain, and paresthesia/dysesthesia. Each item is scored from 0 to 10, with lower scores indicating less impairment. Subscale and total scores are expressed as the mean of the scores. Pre: pre-intervention; HF-TENS: high-frequency transcutaneous electrical nerve stimulation; DM-TENS: dysesthesia-matched transcutaneous electrical nerve stimulation.

	Pre	HF-TENS	DM-TENS
1. Burning	0	0	0
2. Squeezing	7	8	2
3. Pressure	7	0	0
4. During the past 24 hours, your spontaneous pain has been present	5	3	3
5. Electric shocks	8	8	5
6. Stabbing	0	0	0
7. In the past 24 h how many of these pain attacks have you had?	4	3	3
8. Evoked by bruising	10	8	5
9. Evoked by pressure	10	10	5
10. Evoked by sold stimulation	0	9	0
11. Pins and needles	10	10	3
12. Tingling	10	10	5
Superficial spontaneous (1)	0.00	0.00	0.00
Deep spontaneous (2, 3)	7.00	4.00	1.00
Paroxysmal (5, 6)	4.00	4.00	2.50
Evoked (8, 9, 10)	6.67	9.00	3.33
Paresthesia/dysesthesia (11, 12)	10.00	10.00	4.00
Total (1, 2, 3, 5, 6, 8, 9, 10, 11, 12)	6.20	6.30	2.50

**Table 3:** Short-Form McGill Pain Questionnaire version-2 (SF-MPQ2) results before and after each intervention The SF-MPQ2 consists of 22 items and 4 subscales related to continuous pain, intermittent pain, neuropathic pain, and affective descriptors. Each item is scored from 0 to 10, with lower scores indicating less impairment. Subscale and total scores are expressed as the mean of the scores. Pre: pre-intervention; HF-TENS: high-frequency transcutaneous electrical nerve stimulation; DM-TENS: dysesthesia-matched transcutaneous electrical nerve stimulation.

	Pre	HF-TENS	DM-TENS
1. Throbbing pain	0	3	0
2. Shooting pain	9	6	4
3. Stabbing pain	0	0	0
4. Sharp pain	9	3	0
5. Cramping pain	0	0	0
6. Gnawing pain	0	0	0
7. Hot-burning pain	0	0	0
8. Aching pain	0	0	0
9. Heavy pain	0	0	0
10. Tender	5	7	2
11. Splitting pain	0	0	0
12. Tiring-exhausting	0	0	0
13. Sickening	6	5	0
14. Fearful	0	0	0
15. Punishing-cruel	0	0	0
16. Electric-shock pain	3	4	2
17. Cold-freezing pain	0	0	0
18. Piercing	0	0	0
19. Pain caused by light touch	2	4	2
20. Itching	0	0	0
21. Tingling or 'pins and needles'	6	5	3
22. Numbness	7	8	4
Continuous (1, 5, 6, 8, 9, 10)	0.83	1.67	0.33
Intermittent (2, 3, 4, 11, 16, 18)	3.50	2.12	1.00
Neuropathic (7, 17, 19, 20, 21, 22)	2.50	2.83	1.50
Affective (12, 13, 14, 15)	1.50	1.25	0.00
Total (1-22)	2.14	2.05	0.77

**Table 4:** Zurich Claudication Questionnaire (ZCQ) results before and after each intervention The ZCQ consists of 18 items and 3 subscales related to symptoms, function, and satisfaction, with severity scored from 1 to 5, and physical activity and satisfaction scored from 1 to 4, with lower scores indicating less impairment. Subscale and total scores are expressed as the mean of the scores. Pre: pre-intervention; HF-TENS: high-frequency transcutaneous electrical nerve stimulation; DM-TENS: dysesthesia-matched transcutaneous electrical nerve stimulation.

	Pre	HF-TENS	DM-TENS
1. The pain you have on average, including pain in your back, buttocks and pain that goes down the legs?	5	5	3
2. How often have you had back, buttock, or leg pain?	5	5	5
3. The pain in your back or buttocks?	2	2	2
4. The pain in your legs or feet?	5	5	3
5. Numbness or tingling in your legs or feet?	5	5	3
6. Weakness in your legs or feet?	5	5	5
7. Problems with your balance?	5	5	5
8. How far have you been able to walk?	2	2	2
9. Have you taken walks outdoors or in mails for pleasure?	3	3	3
10. Have you been shopping for groceries or other items?	3	3	3
11. Have you walked around the different rooms in your house or apartment?	3	3	3
12. Have you walked from your bedroom to the bathroom?	3	3	3
13. The overall result of back operation?	2	2	2
14. Relief of pain following the operation?	4	3	1
15. Your ability to walk following the operation	4	4	4
16. Your ability to do housework, yard work, or job following the operation?	4	4	4
17. Your strength in the thighs, legs, and feet?	4	4	3
18. Your balance, or steadiness on your feet?	4	4	4
Symptoms (1-7)	4.57	4.57	3.71
Function (8-12)	2.80	2.80	2.80
Satisfaction (13-18)	3.39	3.25	3.20
Total (1-18)	3.78	3.72	3.22

The total NPSI score did not decrease with HF-TENS (6.30) compared to the pre-score (6.20), but a decrease was observed with DM-TENS (2.50). Specifically, the scores for paroxysmal, evoked, and paresthesia/dysesthesia did not decrease with HF-TENS (paroxysmal: 4.00, evoked: 9.00, paresthesia/dysesthesia: 10.00) compared to the pre-scores (paroxysmal: 4.00, evoked: 6.67, paresthesia/dysesthesia: 10.00), whereas they decreased with DM-TENS (paroxysmal: 2.50, evoked: 3.33, paresthesia/dysesthesia: 4.00). The total SF-MPQ2 score decreased with both HF-TENS (2.05) and DM-TENS (0.77) compared to the pre-score (2.14). Specifically, the scores for continuous and neuropathic pain did not decrease with HF-TENS (continuous: 1.67, neuropathic pain: 2.83) compared to the pre-scores (continuous: 0.83, neuropathic pain: 2.50), whereas they decreased with DM-TENS (continuous: 0.33, neuropathic pain: 1.50). The total ZCQ score decreased with both HF-TENS (3.72) and DM-TENS (3.22) compared to the pre score (3.78). Specifically, the symptom score did not decrease with HF-TENS (4.57) compared to the pre-score (4.57), whereas it decreased with DM-TENS (3.71). In addition, the improvement in ZCQ symptom scores showed no decrease from pre (4.57) to HF-TENS (4.57), with a score change of 0.00. However, from HF-TENS (4.57) to DM-TENS (3.71), a reduction of 0.86 was observed, exceeding the MCID (0.84). The improvement in function scores from pre (2.80) to HF-TENS (2.80) was 0.00, and similarly, DM-TENS (2.80) showed no improvement, failing to exceed the MCID (0.76).

The patient's introspection revealed that before the stimulation, she expressed, "The numbness makes it hard to move" and "It feels like there's a membrane covering the plantar surface of my feet, and there is hardly a feeling of touching the ground." After HF-TENS, she expressed, "The numbness has slightly improved," and after DM-TENS, "The numbness is gone, and I can feel the plantar surface of my feet touching the ground."

## Discussion

We conducted DM-TENS on a post-operative DLS patient who had chronic lower limb numbness. In this case, the NRS for numbness before the intervention was 8.00, but after the HF-TENS intervention, the NRS was 6.67 ± 0.47 (mean ± SD), and after the DM-TENS intervention, it was 1.67 ± 0.47 (mean ± SD). VAIOR analysis also indicated that DM-TENS was more effective than HF-TENS in improving numbness. Furthermore, in assessments using questionnaires, scores related to numbness, such as paroxysmal, evoked, and paresthesia/dysesthesia in the NPSI, and continuous and neuropathic pain in the SF-MPQ2, did not decrease with HF-TENS but did show a reduction with DM-TENS.

First, regarding the pathophysiological mechanism of numbness in this case, lumbar degenerative diseases are thought to cause numbness by compressing the nerve roots and cauda equina. However, the mechanism by which numbness persists even after surgical decompression remains unclear. Before the intervention, this case showed improvement in low back pain, but the numbness persisted. It has been reported that unmyelinated C fibers, which transmit thermal and pain sensations, significantly improve six weeks post-operatively, while myelinated Aβ fibers, which transmit numbness and sensory abnormalities, do not show significant improvement within 12 months after surgery [15]. Furthermore, the persistence of numbness is more likely if the condition has lasted for more than a year [16], suggesting that the numbness in this case was difficult to resolve post-operatively. Additionally, prolonged mechanical stimulation can damage Aβ fibers and their axons, leading to neural sensitization and spontaneous ectopic discharges [17]. Moreover, repeated abnormal sensory input may cause hyperexcitability of wide-dynamic-range neurons in the spinal dorsal horn and related brain regions [18], which could also explain why the numbness persisted after surgery in this case.

Next, regarding DM-TENS and HF-TENS, as mentioned earlier, it is important to emphasize that DM-TENS demonstrated significantly greater improvement in numbness compared to HF-TENS. HF-TENS has been used for pain and neuropathic pain, but issues such as the lack of established settings and inconsistent effects have been pointed out [19]. In contrast, DM-TENS individually adjusts the electrical stimulation to match the sensation of numbness, allowing the stimulation to counteract the perception of numbness, which has been reported to improve numbness in patients with spinal cord injury and stroke [9]. Although the affected area in this case of DLS differs from that in previous studies, both share a common pathophysiological mechanism of sensory pathway impairment. In other words, DM-TENS may be effective in individuals whose nerves have undergone plastic changes due to a mismatch between altered peripheral sensory information and the perception of this information in related brain regions, regardless of the site of injury. Considering that 60% of patients experience persistent numbness after lumbar degenerative disease surgery [6] and that numbness contributes to decreased post-operative satisfaction and QOL [6,7], the improvement in numbness observed in this DLS patient represents a significant finding.

This case study has several limitations as a single-case study, making the generalizability of DM-TENS uncertain. While significant improvement was observed in the symptom subscale of the ZCQ, no substantial improvements were seen in other questionnaires or the other subscales of the ZCQ. This may be due to the short follow-up period, which might not have been sufficient to capture meaningful changes, suggesting the need to consider longer intervention periods in future studies. Additionally, although immediate improvement in numbness was observed in this case, previous studies have reported that repeated interventions can lead to cumulative effects and that long-term interventions may enhance the efficacy of DM-TENS [20]. Therefore, future studies should include a longer follow-up period, increase the number of intervention sessions, and consider adjusting the stimulation settings to further enhance treatment outcomes.

## Conclusions

We demonstrated that DM-TENS can improve chronic lower limb numbness that persists after surgical treatment in DLS patients. DM-TENS may also be effective for lower limb numbness in DLS patients. Further research is essential to generalize the use of DM-TENS.
